# Estimating multivariate similarity between neuroimaging datasets with sparse canonical correlation analysis: an application to perfusion imaging

**DOI:** 10.3389/fnins.2015.00366

**Published:** 2015-10-13

**Authors:** Maria J. Rosa, Mitul A. Mehta, Emilio M. Pich, Celine Risterucci, Fernando Zelaya, Antje A. T. S. Reinders, Steve C. R. Williams, Paola Dazzan, Orla M. Doyle, Andre F. Marquand

**Affiliations:** ^1^Centre for Neuroimaging Sciences, Institute of Psychiatry, Psychology and Neuroscience, King's College LondonLondon, UK; ^2^F. Hoffmann-La Roche Ltd.Basel, Switzerland; ^3^Department of Psychosis Studies, Institute of Psychiatry, Psychology and Neuroscience, King's College LondonLondon, UK; ^4^National Institute for Health Research Mental Health Biomedical Research Centre, South London and Maudsley National Health Service Foundation Trust, King's College LondonLondon, UK; ^5^Department of Cognitive Neuroscience, Radboud University Medical Centre, Donders Institute for Brain, Cognition and Behaviour, Radboud UniversityNijmegen, Netherlands

**Keywords:** multivariate analysis, sparse canonical correlation analysis, pharmacological MRI, Arterial Spin Labeling, resting cerebral blood flow, intra-modal data, repeated measures, antipsychotics

## Abstract

An increasing number of neuroimaging studies are based on either combining more than one data modality (inter-modal) or combining more than one measurement from the same modality (intra-modal). To date, most intra-modal studies using multivariate statistics have focused on differences between datasets, for instance relying on classifiers to differentiate between effects in the data. However, to fully characterize these effects, multivariate methods able to measure similarities between datasets are needed. One classical technique for estimating the relationship between two datasets is canonical correlation analysis (CCA). However, in the context of high-dimensional data the application of CCA is extremely challenging. A recent extension of CCA, sparse CCA (SCCA), overcomes this limitation, by regularizing the model parameters while yielding a sparse solution. In this work, we modify SCCA with the aim of facilitating its application to high-dimensional neuroimaging data and finding meaningful multivariate image-to-image correspondences in intra-modal studies. In particular, we show how the optimal subset of variables can be estimated independently and we look at the information encoded in more than one set of SCCA transformations. We illustrate our framework using Arterial Spin Labeling data to investigate multivariate similarities between the effects of two antipsychotic drugs on cerebral blood flow.

## Introduction

Acquiring multiple neuroimaging datasets from the same subject is becoming common practice (Sui et al., 2012). An increasing number of studies are now based on either combining more than one imaging modality [*inter-modal* studies; e.g., Ystad et al. (2011)] or combining more than one measurement from the same modality (*intra-modal* studies; e.g., Elsenbruch et al., 2012). Both provide important benefits: inter-modal studies allow the complementary information from different data modalities to be used whereas intra-modal studies can increase power (e.g., through the use of repeated-measures designs) with respect to single modal studies. To date, intra-modal studies using multivariate methods have focused almost exclusively on differences between datasets, for instance relying on classification algorithms to differentiate between effects in the data.

In the context of pharmacological studies using functional Magnetic Resonance Imaging (phMRI), which commonly employ repeated-measures (i.e., intra-modal) designs, multivariate analyses have exclusively focused on *discriminating* the distributed effects of different drug interventions. Several studies have employed Blood Oxygen-Level Dependent (BOLD)-fMRI for this purpose based on brain activity patterns derived from participants performing a working memory task (Marquand et al., 2011), on functional connectivity based on resting fMRI (Sripada et al., 2013) or in response to drug infusion (Doyle et al., 2013a). Other studies have used Arterial Spin Labeling (ASL) to discriminate the effects of different drugs on regional cerebral blood flow (rCBF) (Chen et al., 2011; Marquand et al., 2012; Doyle et al., 2013b; Paloyelis et al., 2014). This approach offers the advantage over BOLD that the derived measures are quantitative and can be readily compared between scanning sessions.

Although these studies have significantly contributed to our knowledge of the differential effects of pharmacological interventions across brain regions, it is important to identify not only *differences* but also *similarities* between drug effects (Nathan et al., 2014). This is particularly important in drug discovery, where the effects of novel pharmacological compounds are routinely compared to those of existing drugs (Ashburn and Thor, 2004; Wild et al., 2012; Bruns et al., 2015). For these reasons, and in the larger context of intra-modal studies, it would be highly advantageous to develop methods to assess the relationship between sets of measurements (e.g., repeated measures), taking advantage of the multivariate and high-dimensional nature of the data. One classical multivariate technique for estimating the linear relationship between two sets of variables from the same samples (e.g., subjects) is canonical correlation analysis (CCA) (Hotelling, 1936). CCA aims to find a set of linear transformations of the variables in each set of measurements that are maximally correlated with each other. In other words, CCA finds basis vectors (canonical vectors) for each set, such that the correlation between the projections of the data onto these basis vectors is maximized. To find more than one set of basis vectors, CCA can be re-applied to the residual data obtained after subtracting the effect of the first set of canonical vectors from the original data. This way we can look for multivariate relationships in the same data that have not been explained away by the first set of canonical vectors. If the data are normally distributed, CCA is equivalent to maximizing the mutual information between the two datasets (Bach and Jordan, 2003). From a slightly different perspective, CCA can be considered as a method to predict one of the datasets from the other while accounting for the correlations between features (Breiman and Friedman, 1997; Klami et al., 2013).

In the context of high-dimensional neuroimaging studies, the number of variables (voxels) greatly exceeds the number of subjects, rendering the use of CCA for finding image-to-image correspondences challenging. One way to deal with this issue is to use a kernel-based version of CCA, kernel CCA (KCCA, Hardoon et al., 2004). An advantage of KCCA is that it can capture not only linear but also non-linear dependencies between datasets (Cristianini and Shawe-Taylor, 2000; Hardoon et al., 2004). KCCA has been successfully applied to neuroimaging data (Hardoon et al., 2007, 2009), but similarly to classical CCA, this technique yields non-sparse canonical vectors [i.e., the canonical vectors have non-zero coefficients for every variable (e.g., voxel)]. This is not ideal since the main goal in neuroimaging analyses is often to identify brain regions or networks that are most relevant to the question being studied.

With this goal in mind we seek a sparse solution of CCA, Sparse CCA (SCCA). This can be achieved by regularizing CCA with a penalty that imposes sparsity on the entries of the canonical vectors, as proposed by Witten et al. (2009). SCCA finds a sparse linear combination of the variables in one set of measurements that maximally correlates with a sparse linear combination of the variables in the other set of measurements. The corresponding patterns of non-zero coefficients can be interpreted in terms of networks of brain regions that show similar effects across datasets. Due to the penalization term, SCCA can be applied to high-dimensional/small sampled datasets. A sparse version of KCCA has also been developed but sparsity is imposed only on one of the datasets (Hardoon and Shawe-Taylor, 2011).

In neuroimaging there are several lines of related work. SCCA has been used to find multivariate relationships between high-dimensional brain data and low-dimensional genetic, clinical, and/or cognitive measurements (Boutte and Liu, 2010; Le Floch et al., 2012; Lin et al., 2013a, 2014; Avants et al., 2014; McMillan et al., 2014). To our knowledge, the only work to date where SCCA has been used to look at image-to-image multivariate similarities between whole brain datasets is Avants et al. (2010). The authors used SCCA to relate information encoded in different brain imaging modalities aiming to map the relationship between whole-brain changes in cortical thickness (measured with structural MRI) and white matter integrity (measured with diffusion tensor imaging) in dementia patients relative to healthy controls. The authors chose to use a fixed degree of sparsity that yielded a solution with 50% of the variables in each set of measurements equal to zero. Multi-modal data fusion is another important application for CCA and other related methods (see Biessmann et al., 2011 and Sui et al., 2012 for reviews). These methods include variants of partial least squares (PLS), such as multi-way PLS (Martínez-Montes et al., 2004) and variants of independent component analysis (ICA) such as linked ICA (Groves et al., 2011).

This work makes three main contributions. First, the applications of CCA to repeated measures designs and to ASL are novel. Second, we extend the SCCA framework developed by Witten and Tibshirani (2009) to estimate the optimal subset of variables for each imaging set independently, using permutation. This is important because there is no guarantee that each set of imaging measurements (each “view”) has the same properties. For example, one view may have many more activated voxels than the other or may have different smoothness. This is particularly important in drug studies, because different drugs can have very different influences on BOLD activity or rCBF. Optimizing a separate parameter for each view provides the model with additional flexibility to accommodate such differences. A simple simulation is provided to illustrate this effect in the Supplementary Material. Third, we explicitly optimize model parameters controlling sparsity, which is in contrast to most other applications that use a fixed sparsity level (e.g., Avants et al., 2010; McMillan et al., 2014). Finally, we map the information encoded in more than one set of SCCA components, which is in contrast to other applications that only examine the first component. We illustrate the feasibility and utility of our framework using Arterial Spin Labeling data from a neuropharmacological study, with the aim of investigating multivariate similarities between the effects of two antipsychotic drugs (haloperidol and aripiprazole) on resting rCBF in the healthy human brain.

## Materials and methods

### Canonical correlation analysis (CCA)

CCA was first introduced by Hotelling (1936) and is a classical multivariate statistical technique for finding linear relationships between two sets of variables. We begin with two datasets represented by the matrices *X*_1_ and *X*_2_, having dimensions *n* × *p*_1_ and *n* × *p*_2_ respectively, where *n* is the number of samples (e.g., subjects) and *p*_1_and *p*_2_ are the number of variables (e.g., voxels) in set *X*_1_ and *X*_2_, respectively. CCA seeks linear transformations of *X*_1_ and *X*_2_ that are maximally correlated with each other:

$$\begin{array}{l} max_{u,v}q=u^{T}X_{1}^{T}X_{2}v\\ \text{subject to }u^{T}X_{1}^{T}X_{1}u=1\text{ and }v^{T}X_{2}^{T}X_{2}v=1. \end{array}$$

CCA assumes that the columns of *X*_1_ and *X*_2_ are standardized to have mean of zero and standard deviation of one. The vectors *u* and *v*, with dimensions *p*_1_ × 1 and *p*_2_ × 1, respectively, are known as the *canonical vectors* (or weights); the vectors *X*_1_*u* and *X*_2_*v*, with dimensions *n*×1, are known as the *canonical variables*; and *q* is called the *canonical correlation*. A schematic representation of CCA is presented in the Supplementary Material. The solution to the above equation can be obtained analytically (Hotelling, 1936). However, when the number of variables exceeds the number of samples, which is often the case in the context of neuroimaging data, CCA cannot be applied directly, because the analytical solutions require inverting covariance matrices that are rank deficient (singular) in this setting. To circumvent this issue, various authors (Waaijenborg et al., 2008; Parkhomenko et al., 2009; Witten et al., 2009) have proposed a regularized version of CCA, called sparse canonical correlation analysis (SCCA). In this paper, we extend the approach developed by Witten et al. (2009).

### Sparse canonical correlation analysis (SCCA)

Similarly to CCA, SCCA looks for a linear combination of the variables in *X*_1_ that is maximally correlated with a linear combination of the variables in *X*_2_. However, in SCCA two penalty function terms, *P*_1_ and *P*_2_, are introduced to regularize the solution of *u* and *v*:

$$\begin{array}{l} {max}_{u,v}q=u^{T}X_{1}^{T}X_{2}v\\ \text{subject to }{{∥u∥}_{2}}^{2}\leq 1,{{∥v∥}_{2}}^{2}\leq 1\text{ and }P_{1}\left(u\right)\leq c_{1},P_{2}\left(v\right)\leq c_{2}. \end{array}$$

*P*_1_ and *P*_2_ are in general chosen to yield sparse *u* and *v* vectors in that some elements (here, voxel weights) are zero. The constants *c*_1_ and *c*_2_, are regularization parameters, and can be optimized from the data. For sparse norms, they control the amount of sparsity (number of zero elements) in the solution of *u* and *v*. The terms ${{∥u∥}_{2}}^{2}=1$ and ${{∥v∥}_{2}}^{2}=1$ result from replacing the covariance matrix of each dataset by its diagonal, known as “diagonal penalized CCA” (Witten et al., 2009). This simplification has been shown to produce good results in high dimensional classification problems, even when correlation between variables is evident (Dudoit et al., 2002; Tibshirani et al., 2003). In addition, assuming that *X*_1_ and *X*_2_ have been standardized, as described before, the solution of SCCA is unique, i.e., the vectors *u* and *v* are unique even when the number of variables in each dataset, *p*_1_ and *p*_2_, greatly exceed the number of samples, *n*. In this paper, we use the Least Absolute Shrinkage and Selection Operator (LASSO) penalty function (Tibshirani, 1996) for both *u* and *v* vectors: *P*_1_(*u*) = ∥*u*∥_1_, and *P*_2_(*v*) = ∥*v*∥_1_ where ∥*u*∥_1_and ∥*v*∥_1_are the L1-norms of vector *u* and *v*, respectively (i.e., the sum of the absolute value of the elements of each vector). The estimation procedure for SCCA proposed by Witten et al. (2009) is based on a penalized matrix decomposition and is summarized in the Supplementary Material. We note that although other penalty functions are possible (such as the total variation penalty), the LASSO penalty performs well in practice for neuroimaging data. In practice, the solutions obtained by SCCA are less sparse than for other problems such as logistic regression because of the additional L2 penalty implicitly imposed by the penalized matrix decomposition (see Witten et al., 2009). Additionally, we restrict the elements of *u* and *v* to be nonnegative (i.e., *u*_*i*_, *v*_*i*_ = 0), so that the solution of SCCA can be interpreted as a sparse weighted average of the variables in *X*_1_ that maximally correlates with a sparse weighted average of the variables in *X*_2_ (Witten and Tibshirani, 2009). It is important to note here that SCCA does not provide a variable-wise (e.g., voxel-wise) measure of similarity between the two datasets. It provides a single measure of similarity (the canonical correlation) and a vector of weights, representing the basis vectors *u* and *v*, for each dataset.

#### Selection of regularization parameters

We use a permutation-based approach for choosing the regularization parameters from the data. We adapt the permutation-based framework proposed by Witten and Tibshirani (2009) in order to allow the regularization parameter for each set of variables to be optimized independently of the other set. In other words, we allow a different value for *c*_1_and *c*_2_. In the original implementation, this is not allowed and the same parameter setting must be used for both. As noted, this is important because *X*_1_ and *X*_2_ may not have the same properties (e.g., number of activated voxels). This does, however, entail estimating two model hyper parameters in place of one. A didactic simulation illustrating this is presented in the Supplementary Material. Since we use SCCA with the L1-norm penalty function, as mentioned above, these parameters control how sparse the solution is, i.e., how many elements of *u* and *v* are zero. The permutation-based algorithm proceeds as follows:

1. Given a value of *c*_1_ and *c*_2_, we independently permute the samples of the datasets, *X*_1_ and *X*_2_, to create two new datasets, $\dot{X_{1}}$ and $\dot{X_{2}}$. We then apply SCCA with parameters *c*_1_ and *c*_2_ as described before, to obtain vectors $\dot{u}$ and $\dot{v}$.
2. We calculate the canonical correlation for the permuted data: $\dot{q}=Corr\left(\dot{X_{1}}\dot{u},\;\dot{X_{2}}\dot{v}\right)$.
3. We repeat steps 1 and 2 k times, each time by permuting the data again and calculating a new $\dot{q}$.
4. We repeat steps 1 to 3 but use a different choice of parameters *c*_1_ and *c*_2_ (a grid-search approach is used to exhaustively cover all combinations of *c*_1_ and *c*_2_).
5. For each pair of parameters *c*_1_ and *c*_2_ we then use the Fisher transformation to convert the correlation values, $\dot{q}$, into random variables that are approximately normally distributed and compute a *z*-statistic: $z_{c_{1}c_{2}}=\left(Fisher\left(q\right)-mean\left(Fisher\left(\dot{q}\right)\right)\right)∕std\left(Fisher\left(\dot{q}\right)\right)$, where *q* is the canonical correlation obtained using the original data and parameters *c*_1_ and *c*_2_. We then choose *c*_1_ and *c*_2_ that yield the highest *z*-statistic.
6. Finally, we re-estimate SCCA using the original data and the chosen parameters *c*_1_ and *c*_2_ from the last step to obtain the final *u* and *v* vectors.

It is common practice in machine learning and statistics to estimate a model using the entire dataset once model selection has been performed using the permutation-based approach described above or other method, such as cross-validation (Burbidge et al., 2001; Lin et al., 2013b). The permutation-based approach is preferable to cross-validation when the number of samples is small, since it avoids the need to split the data into even smaller sets for training and testing (Witten and Tibshirani, 2009). For this reason and the fact that our approach is purely unsupervised, we do not separate the data into training and test sets in this study.

#### Determination of significance

To determine the significance of the estimated canonical correlation values (i.e., to measure how unlikely it is to obtain a particular value of *q* or higher if there was no relationship between the two datasets) we use the same permutation-based approach described above. This involves counting how many times the canonical correlations obtained with the permuted data, $\dot{q}$, are equal or higher to the canonical correlation obtained with the original data, *q*, and dividing by the number of permutations. This calculation yields an estimate of the *p*-value for the overall significance of the correlation.

#### Multiple canonical vectors

The algorithm for SCCA described above can be extended to output more than one set of canonical vectors. The procedure to obtain the i^th^ set of canonical vectors simply involves applying the algorithm to the residual data obtained from removing the effect of the i^th^-1 set of canonical vectors from the data. This technique is known in linear algebra as matrix deflation. For example, the second set of canonical vectors is obtained by applying SCCA to the dataset:

$$\begin{array}{l} {{X^{\prime }}_{1}^{T}X^{\prime }}_{2}\leftarrow \;{X_{1}^{T}X}_{2}-\left(u^{T}{X_{1}^{T}X}_{2}v\right)uv^{T}, \end{array}$$

where *u* and *v* are the first set of canonical vectors. It is important to note here that the different sets of vectors are not orthogonal given the presence of the penalty terms in the main SCCA equation, although alternative approaches to deflation can be employed to enforce orthogonality (Monteiro et al., 2014).

### Pharmacological MRI data

We illustrate SCCA with Arterial Spin Labeling (ASL) data published in Handley et al. (2013). This study focused on univariate differences between the effects of two antipsychotic drugs (haloperidol and aripiprazole) on resting cerebral blood flow (rCBF) in the human brain. The details of the study can be found in Handley et al. (2013), but for completeness, the main experimental details are reported briefly here.

#### Participants

Twenty healthy right-handed English speaking Caucasian males, aged 18–33 (mean 23 years SD 4.5), participated in the experiment. Mean IQ and body mass index were within the normal range (Handley et al., 2013). Participants were nonsmoking, university students with no recent or current drug or medication use and had no exposure to psychotropic medication, nor a history of personal or familial psychiatric diagnosis. Written informed consent was obtained from all participants and the study was approved by the Human Research Ethics Committee of the Institute of Psychiatry, London, and conducted in compliance with the Declaration of Helsinki.

#### Design

A crossover, randomized within-subject, double blinded, placebo-controlled design was used. Participants received a single oral dose of haloperidol (3 mg), aripiprazole (10 mg), or placebo, presented in identical capsules, in a randomized order on three visits. Antipsychotic dose and time of administration (3 h and 30 min) before scanning were intended to achieve a striatal D2 receptor occupancy level comparable across the two compounds (Handley et al., 2013). A minimum of 14 days separated each visit to allow for drug washout. No alcohol or medications were used for 24 h, nor caffeine for 6 h, before scanning.

#### Data acquisition

Participants were scanned with their eyes open and images were acquired in a General Electric Signa HDX 1.5 T scanner at the Centre for Neuroimaging Sciences, Institute of Psychiatry. Regional CBF measurements were made using a pulsed-continuous Arterial Spin Labeling technique (pCASL, Dai et al., 2008). The pulse sequence parameters can be found in Handley et al. (2013).

After labeling, images were acquired with a three-dimensional Fast Spin Echo (FSE) spiral multishot readout. To minimize blurring, the spiral acquisition for each slice was very short (4 ms), and the required resolution was achieved with eight interleaves (TE 32 ms/TR = 5500 ms; ETL = 64). Images were acquired at a 48 × 64 × 48 matrix on an 18 × 24 × 18 cm field of view. Images were reconstructed to a 256 × 256 matrix, resulting in a nominal spatial resolution of 1 × 1 mm in plane. Sixty slices of 3 mm thickness were obtained. Three pairs of tagged-untagged images were collected. Following the three ASL control-label pair averages, images were acquired with the same imaging sequence but with inversion recovery preparation instead of ASL. These images were used to quantify blood flow from the ASL, as described in Handley et al. (2013). Two additional structural, high spatial resolution images were acquired for co-registration and normalization, including a T2 weighted fast spin echo and fluid-attenuated inversion-recovery fast spin echo scan to exclude the presence of any brain pathology.

#### Data analysis

The pre-processing of the ASL data was performed according to Handley et al. (2013). The rCBF images were processed using Statistical Parametric Mapping 8 (SPM8, www.fil.ion.ucl.ac.uk/spm). For each participant: (i) the extra-cerebral signal from the T2 scan was removed using the “Brain Extraction Tool” (BET) of the Functional Software Library (FSL, Smith, 2002). The skull stripped T2 volume and its corresponding binary mask were then coregistered to the rCBF map; (ii) the coregistered, binary, brain-only mask, was multiplied by the rCBF map to remove extra-cerebral signal from this scan. The skull stripped T2 and rCBF maps were then coregistered back to the space of the original T2 scan; (iii) the original T2 scan was normalized to the MNI based T2 template provided in SPM, and the transformation matrix was applied to the rCBF map and the T2 scan. After normalization, the data were downsampled to 3 × 3 × 3 mm voxels and then smoothed using a 10 mm Gaussian smoothing kernel.

In addition, we mean scaled each ASL scan (i.e., subtracted the mean of each image) to remove the session effect and subtracted the placebo scan from the haloperidol and aripiprazole images for each subject individually. This way we look for similarities in the mean effects of haloperidol and aripiprazole relative to placebo. The resulting images were then masked with a gray matter template (obtained by thresholding the gray matter template provided by SPM) and used as input to SCCA. Finally, SCCA analyses were based on 18 subjects per group (drug) as the data of two subjects had to be discarded because these subjects developed nausea and vomiting with aripiprazole.

## Results

**Figure 1:**
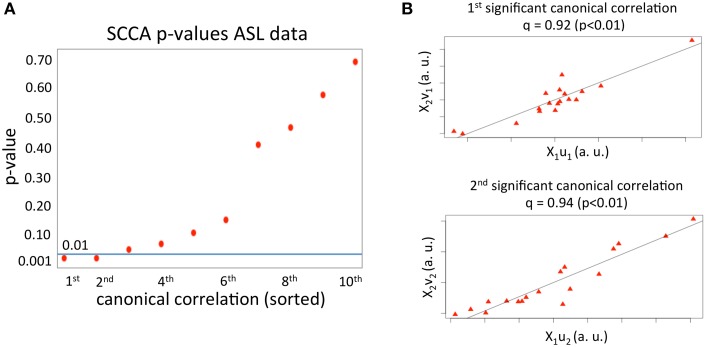
**(A)** *p*-values for the first 10 canonical correlations obtained from applying SCCA to the ASL dataset. The horizontal line depicts the *p* = 0.01 value. The *x* axis comprises the order of the canonical correlations. **(B)** Correlation between the first and second set of canonical variables.

We first applied SCCA to the haloperidol (*n* = 18 subjects and *p*_1_ = 37702 variables) and aripiprazole data (*n* = 18 subjects and *p*_2_ = 37702 variables) to find the first 10 canonical correlations and sets of canonical vectors, as described above. As can be seen in Figure 1A, only two correlations had a *p* < 0.01 (*p*-value estimated using 1000 permutations). The first significant canonical correlation was 0.92 (*p*-value = 0.003) and the second significant correlation was 0.94 (*p*-value = 0.003). The value of the regularization parameters, *c*_1, 2_, that account for how sparse the solution vectors are was varied from 0.3 to 0.9 in steps of 0.1. This range was chosen based on pilot runs, and covered a wide range of sparsity values while enforcing sufficient regularization to avoid the trivial solution (Hardoon et al., 2004). The optimal parameters (*c*_1_ = 0.4 and *c*_2_ = 0.7) were chosen based on the first set of canonical vectors and using 1000 permutations, as described in the Materials and Methods section.

The first two sets of significant canonical variables are plotted in Figure 1B. As can be seen, the correlation between canonical variables (linear transformation of the original samples) is very high, meaning that in the space spanned by the first and second significant set of canonical vectors, the mean effects of the two drugs on rCBF are highly correlated.

**Figure 2:**
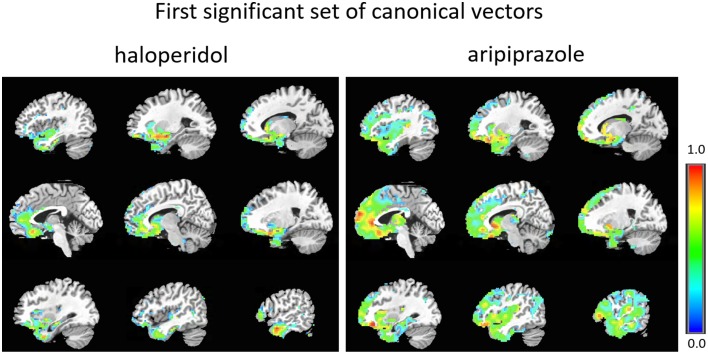
**First set of significant canonical vectors for haloperidol and aripiprazole**. The canonical vectors have the same dimension as the number of variables (in this case voxels) and can therefore be represented in the original voxel space as an image. The weights (entries of the canonical vectors) are all positive by construction.

**Figure 3:**
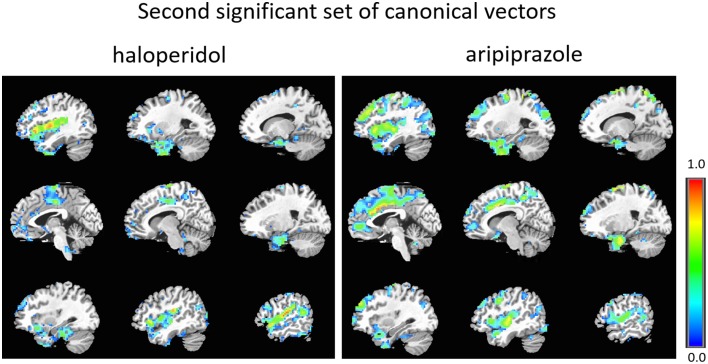
**Second set of significant canonical vectors for haloperidol and aripiprazole**. The canonical vectors have the same dimension as the number of variables (in this case voxels) and can therefore be represented in the original voxel space as an image. The weights (entries of the canonical vectors) are all positive by construction.

The first set of canonical vectors with significant correlation is shown in Figure 2. As can be seen, the first set of canonical vectors is more sparse for haloperidol relative to aripiprazole but with many overlapping regions between the drugs. In both cases, most clusters of non-zero variables were distributed across areas of the frontal cortex (especially medially), limbic system and the striatum. The second set of canonical vectors with significant correlation were characterized by clusters of non-zero variables distributed across areas of the temporal cortex, insula and the middle part of the cingulum (Figure 3). We also present an alternative visualization of the weights in the Supplementary Material. As can be seen in Figure 2 and in the Supplementary Material, the rCBF signature obtained from the first set of canonical vectors shows a similar pattern of non-zero coefficients (network) for haloperidol and aripiprazole on rCBF across the brain. Both drugs seem to affect a network of regions that mainly comprises frontal regions (such as the orbito-frontal cortex and olfactory cortex), the striatum structures (caudate and putamen), the amygdala, anterior cingulate cortex, straight gyrus, the insula, and the temporal poles. One of the main differences between the networks highlighted for the two drugs seems to be the extent of non-zero weight found in frontal regions, which is much larger for aripiprazole (Figure 2).

As can be seen, the second set of vectors (Figure 3 and Supplementary Material) highlights similar networks for the two drugs, comprising mostly temporal regions (temporal poles, superior, and middle temporal cortex), and Heschl's gyrus, the insula, the amygdala, parahippocampal regions, the frontal operculum and the supra-marginal cortex.

## Discussion

Here we illustrated the utility of SCCA for investigating multivariate linear relationships between high-dimensional intra-modal neuroimaging datasets acquired for the same group of subjects. We applied the technique to pharmacological ASL data acquired using a repeated measures design to study the effects of two antipsychotic drugs [haloperidol—first-generation (FGA), and aripiprazole—third-generation, (Mailman and Murthy, 2010); on rCBF of healthy volunteers]. We found similar distributed rCBF networks that maximize the correlation between the two drugs. In particular, the first set of canonical vectors highlighted a network comprising mainly the striatum (caudate and putamen), anterior cingulate cortex, orbital frontal cortex, amygdala, straight gyrus (rectus), olfactory cortex, and temporal poles for both antipsychotics. The second set of canonical vectors highlighted a network comprising mostly temporal regions, the insula, frontal operculum, the supra-marginal cortex and Heschl gyrus. Differences between the networks obtained from the first set of canonical vectors highlight a larger number of frontal regions involved in the effect of aripiprazole compared to haloperidol. The second set of vectors suggests a stronger response in the cingulum again in aripiprazole compared to haloperidol. These differences could be related to the fact that haloperidol is principally a dopamine D2, D3, and D4 receptor antagonist (Okuyama et al., 1997), while aripiprazole on the other hand is a partial agonist at D2 receptors, a serotonin 5-HT1a partial agonist, and an antagonist at serotonin 5-HT2a receptors (Pae et al., 2008).

It is not straightforward to compare these results with previous univariate analyses of the same data, because the SCCA coefficients have a different interpretation to mass-univariate general linear model (GLM) coefficients: SCCA coefficients are multivariate and describe the weight applied to each brain region to maximize the overall correlation between the inputs. In contrast, GLM coefficients describe focal differences between brain regions in a univariate sense. Nevertheless, many features of the networks detected do seem to overlap with previous findings (Handley et al., 2013). Handley et al. (2013) have shown that both haloperidol and aripiprazole have focal effects on rCBF in the striatum, frontal regions, insula, hippocampus, and cingulum. Univariate differences between the two drugs were mostly found in the motor cortex, where changes in rCBF were present for haloperidol but not aripiprazole, and vice-versa in the cerebellum and occipital regions. The effects observed in the motor cortex for haloperidol, could be due to the fact that, in contrast to aripiprazole, haloperidol induces Parkinsonian-like symptoms (or extrapyramidal symptoms) that start to be detectable at the dose used in Handley et al. (2013) and in the present study. Primary somato-motor cortex and inferior parietal cortex activation can be seen in akinetic Parkinson's disease patients (Sabatini et al., 2000). Although we do not observe the motor cortex as part of the network obtained for haloperidol, differences to previous studies could be due to the fact that we are examining similarities between whole-brain effects of the two drugs on rCBF, rather than differences in focal effects. In other words, this could simply reflect different information conveyed by the univariate and multivariate analyses, including the different statistical methodologies used in each approach.

Other univariate pharmacological studies using FGA, second/third generation antipsychotics or both types have shown significant effects in striatal functional activation (Barouche et al., 1994; Miller et al., 2001; Lahti et al., 2003, 2009; Kim et al., 2008). Both types of drugs have also been found to affect the temporal and frontal cortices (Barouche et al., 1994; Bartlett et al., 1996; Lahti et al., 2005, 2009; Kim et al., 2008), as well as the cingulum (Lahti et al., 2005; Kim et al., 2008). In interpreting the results presented here, it is important to keep in mind that they reflect the particular drug doses administered. Different doses of either drug may result in different patterns of findings. This issue also affects univariate analyses.

The framework presented here is highly flexible and can easily accommodate more than two sets of variables (Witten et al., 2009). This is potentially useful for studies with more than two repeated measures, such as more than two compounds of the same pharmacological class, or imaging modalities. In addition, SCCA can be used in a supervised context, where in addition to the sets of imaging measurements we have an output variable that we would like to predict, such as a cognitive variable or a clinical score. Supervised SCCA seeks linear combinations of variables in each set that are highly correlated with each other and associated with the outcome variable (Witten et al., 2009). Sparse CCA can also be easily extended to incorporate other regularization functions, such as a group-structured penalty function (Chen et al., 2012; Lin et al., 2014). Our results indicate that the LASSO penalty we employed in this work performed relatively well in finding an optimal number of variables to maximize the canonical correlation between drug conditions. Nevertheless, an interesting area of future work could be to evaluate alternative structured sparse regularization penalties such as total variation (Michel et al., 2011) or GraphNet (Grosenick et al., 2013). These may be useful to accommodate spatial dependencies between brain voxels (for example smoothness), and may therefore be better suited to modeling the anatomical structure of neuroimaging data than the penalty used here.

Here we used a permutation-based approach for choosing the optimal sparsity parameters from the data. Alternative data-driven ways of choosing these parameters could be investigated such as cross-validation approaches for larger sample-sized data and other criteria for sparsity optimization, such as stability and reproducibility of the obtained patterns (Meinshausen and Bühlmann, 2010; Ryali et al., 2012). Bayesian techniques may be particularly useful in this regard as they provide the ability to automatically infer regularization parameters from the data. Indeed, a Bayesian CCA approach has recently been proposed (Klami et al., 2013) although this does not permit sparsity over the feature (i.e., voxel) weights. Moreover, this approach has been demonstrated to show inferior performance for detecting associations between high-dimensional neuroimaging data and genetic polymorphisms (Grellman et al., 2015). For future work, it may be interesting to extend Bayesian methods to provide sparsity over the features, perhaps using a similar approach to the Bayesian LASSO (Park, 2005) but at the present time we favor the sparse CCA approach presented here.

Sparse CCA has many other potential uses in neuroimaging in addition to the application reported here. The canonical variables can be compared between groups (e.g., between patients and control subjects) when there are more than one group of subjects for whom more than one dataset was acquired (Sui et al., 2012). Sparse CCA can also be used as a feature selection method in a discriminative framework. In other words, SCCA can be used to discriminate two or more classes based on variables that maximize the multivariate correlation between two sets of measurements obtained for each class. This may be useful to find an optimal subspace for classification based on multiple measurements where class differences are more salient.

One limitation of the methodological approach presented here is the fact that even though we constrain the entries of the canonical vectors to be non-negative in order to facilitate their interpretation (in terms of a weighted average of the variables in each dataset), these coefficients are part of a multivariate model and, consequently, regional inferences cannot be performed directly on SCCA maps (i.e., canonical vectors should not be thresholded for local inference). This issue is not specific to SCCA and affects classical CCA, KCCA, and most multivariate-based predictive models used in neuroimaging, such as commonly used linear classifiers. The only way to mitigate this problem to some extent, without relying on *post-hoc* permutation tests and corresponding analytical approximations (Gaonkar and Davatzikos, 2013), is to enforce sparsity within the model, as was done in this work.

To our knowledge this is the first study to present multivariate similarity measures of the effects of two antipsychotic drugs on human rCBF. In general, these measures can be potentially useful to characterize the effects of any two different tasks or conditions (such as mental and neurological disorders) on human brain activity. In drug studies, similarities are particularly important in drug discovery, where the effects of novel pharmacological compounds are routinely compared to those of existing drugs.

To conclude, we have shown that SCCA is a powerful technique to investigate multivariate similarities between different sets of measurements. In particular, we have demonstrated its feasibility when applied to the challenging question of finding meaningful image-to-image correspondences between neuroimaging data acquired from the same group of subjects. Using ASL data from two different classes of drugs, the feasibility was clear, even in the case of small sample sizes, typical of pharmacological imaging studies.

### Conflict of interest statement

The authors declare that the research was conducted in the absence of any commercial or financial relationships that could be construed as a potential conflict of interest.
