# Supplementary material for: Estimating multivariate similarity between neuroimaging datasets with sparse canonical correlation analysis: an application to perfusion imaging
Source: Front Neurosci. 2015 Oct 13;9:366. doi: 10.3389/fnins.2015.00366 (PMC4603249; doi:10.3389/fnins.2015.00366)
Supplement: Supplementary file 1 [file DataSheet1.PDF]

Supplementary material for:

Estimating multivariate similarity between neuroimaging datasets with sparse canonical correlation analysis: an application to perfusion imaging

---

Maria J. Rosa[1], Mitul A. Mehta[1], Emilio M. Pich[2], Celine Risterucci[2], Fernando Zelaya[1], A. A. T. Simone Reinders[3], Steve C. R. Williams[1], Paola Dazzan[3,4], Orla M. Doyle[1*] and Andre F. Marquand[5,1*]

[1]Centre for Neuroimaging Sciences, Institute of Psychiatry, Psychology & Neuroscience, King's College London, London, UK.

[2]F. Hoffmann-La Roche Ltd., Basel, Switzerland.

[3]Department of Psychosis Studies, Institute of Psychiatry, Psychology & Neuroscience, King's College London, London, UK.

[4]National Institute for Health Research Mental Health Biomedical Research Centre, South London and Maudsley National Health Service Foundation Trust, King's College London, London, England.

[5]Donders Institute for Brain, Cognition and Behaviour. Radboud University, Nijmegen, The Netherlands.

[*]Joint senior authors

Supplementary Methods

Introduction to Canonical Correlation analysis.

A schematic overview, showing the application of CCA to neuroimaging data is provided in Figure S1

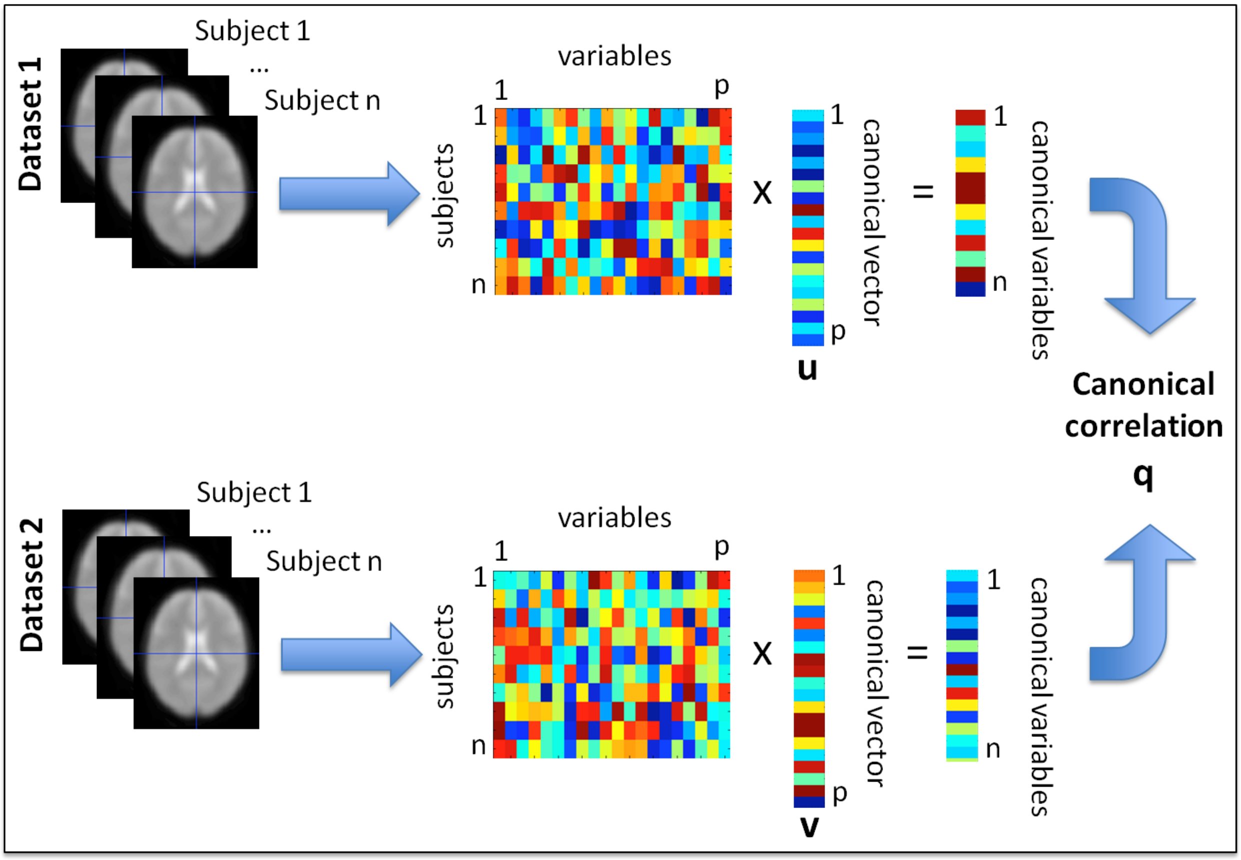

Figure S1: Overview SCCA applied to neuroimaging data

As noted in the main text, SCCA is estimated using a penalised matrix decomposition (see Witten et al, 2009) for full details. This procedure can be summarized in Algorithm S1 below:

Algorithm S1: SCCA

Input: $X_1 \in \mathbb{R}^{n \times p_1}, X_2 \in \mathbb{R}^{n \times p_2}, c_1 \in \mathbb{R}, c_2 \in \mathbb{R}$.

Output: $u \in \mathbb{R}^{p_1 \times 1}, v \in \mathbb{R}^{p_2 \times 1}, q \in \mathbb{R}$.

1. Initialize v to have L2-norm equal to 1.
2. Iterate until convergence:
    a. $v \leftarrow \arg max_v\, u^T X_1^T X_2 v$ subject to $\|v\|_2{}^2 \leq 1, v_i \geq 0$ and $P_2(v) \leq c_2$.
    b. $u \leftarrow \arg max_u\, u^T X_1^T X_2 v$ subject to $\|u\|_2{}^2 \leq 1, u_i \geq 0$ and $P_1(u) \leq c_1$.

Steps a. and b. can be solved using a soft-thresholding operator, $S(a,b) = sgn(a)(|a| - c)_+$, as described in full detail in Witten and Tibshirani (2009): $u \leftarrow \frac{S\left(\left(X_1^T X_2 v\right)_+, \Delta_1\right)}{\left\|S\left(\left(X_1^T X_2 v\right)_+, \Delta_1\right)\right\|_2}$, where $\Delta_1 = 0$ if $\|u\|_1 \leq c_1$; otherwise $\Delta_1 > 0$ is chosen so that $\|u\|_1 = c_1$. We use a similar update rule for v.

3. $q \leftarrow corr(X_1 u, X_2 v)$.

Simulated data to illustrate separate regularization parameters

We simulated a simple example dataset to illustrate the benefit of optimizing separate regularization parameters in SCCA. Note that this simulation is principally didactic; we do not intend it to provide an accurate representation of the expected noise properties of neuroimaging data. This example comprises two views of the same data (dataset X_1 and dataset X_2), with p_1 and $p_2 > n$. These could be two different data modalities (e.g. structural MRI and DTI), or two different measures from the same modality (e.g. repeated measures of rCBF under different psychopharmacological challenges). We also include two scenarios depending on whether the datasets are balanced across views. That is, depending on whether the number of non-zero coefficients were the same (balanced) or different (unbalanced). This simulates the scenario where one view may have a larger number of activated voxels relative to the other. We included $n = 20$ subjects and $p_1 = p_2 = p = 1000$ variables and each dataset was generated using two latent sources (denoted by u_1 and u_2 for dataset X_1 and by v_1 and v_2 for dataset X_2). For the balanced case, data were generated according to the following procedure:

1. Let u_1 be a vector of length 1000, with 100 ones and 900 zeros;
2. Let u_2 be a vector of length 1000, with 900 zeros and 100 ones;
3. Let v_1 be a vector of length 1000, with 50 zeros, 50 ones, 200 zeros, 100 ones, and 600 zeros;
4. Let v_2 be a vector of length 1000, with 850 zeros, 100 ones, and 50 zeros;

5. Let w_1 and w_2 be two orthogonal vectors of length 20, with 10 ones and 10 zeros, and 10 zeros and 10 ones, respectively.

The data matrices X_1 and X_2 are then generated as follows:

1. $X_1 = w_1 u_1^T + w_2 u_2^T + \mathcal{N}(0,0.5)$;
2. $X_2 = w_1 v_1^T + w_2 v_2^T + \mathcal{N}(0,0.5)$;

where $\mathcal{N}(0,0.5)$ represents a Gaussian noise component. The data generation procedure for the unbalanced case was identical except the number of non-zero coefficients was increased for the first source in the first view and decreased in the first source for the second view. That is, the following modifications were made to the data generation procedure:

1. Let u_1 be a vector of length 1000, with 100 ones, 300 zeros, 400 ones and 200 zeros;

...

3. Let v_1 be a vector of length 1000, with 90 zeros, 10 ones, 200 zeros, 100 ones, and 600 zeros;

...

The data generation procedures for both cases is shown graphically in Figure S2.

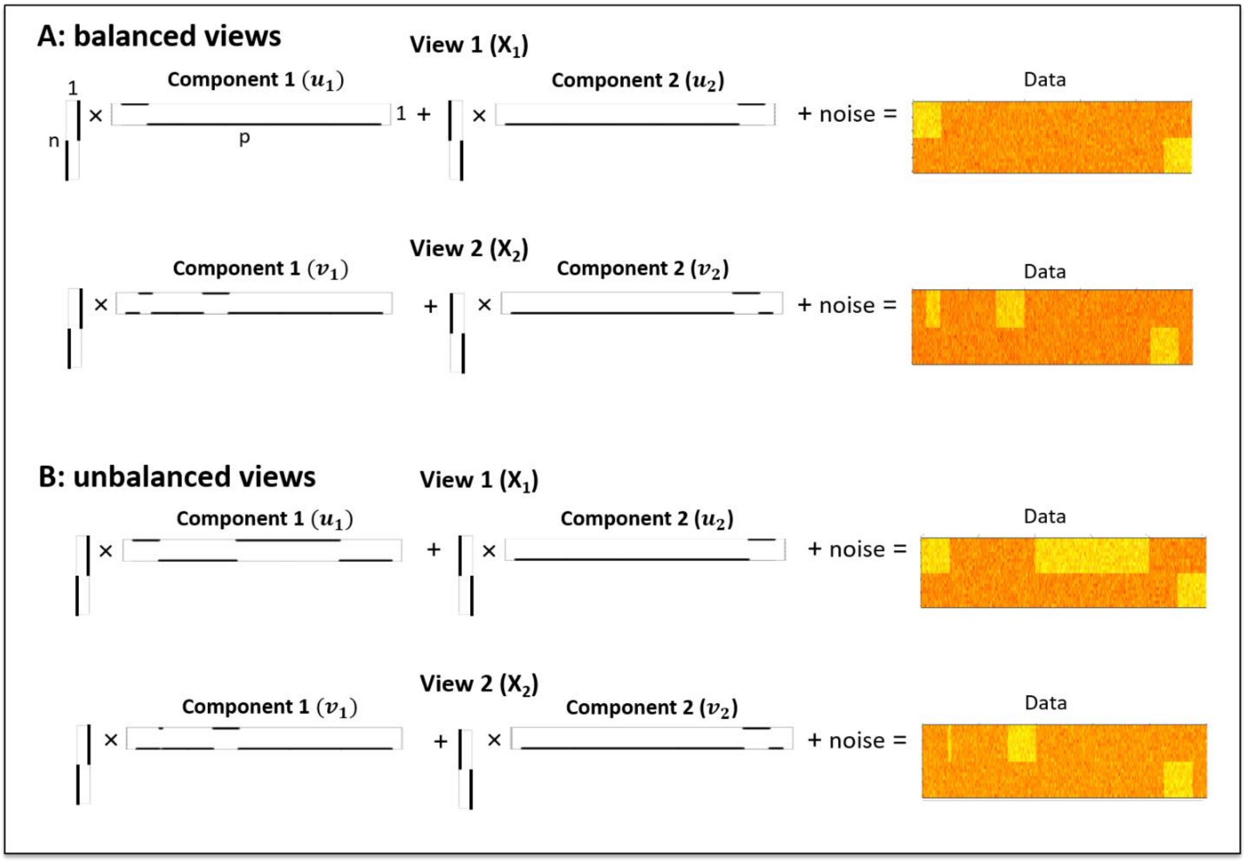

Figure S2: Simulated data generation procedure, showing the creation of two views of the data (X_1 and X_2), each having two components (u_1, u_2 to construct X_1 and v_1 and v_2 to construct X_2). These are each constructed by forming an outer product with a vector of subject loadings. There are also two examples of each dataset, one where the number of non-zero features is the same across views (balanced) and one where the number of non-zero features is different (unbalanced). The dimensions of each vector are shown for the first example.

Supplementary Results

Simulated data

We begin by linearly transforming the toy example data matrices, X_1 and X_2, using vectors u and v with 1000 ones each (this corresponds to summing the variables in each sample weighted equally). The correlation between samples (subjects) of the two datasets is 0.18 for the balanced case and 0.01 for the unbalanced case. If we now use SCCA to find new linear transformations (weighted sum of the variables

in each sample), called canonical variables, the correlation, also known as canonical correlation, is now > 0.99 in both cases (p-value < 0.01). To illustrate the effect of the different parameter optimization procedures, we plot the first weight vector under two optimization schemes. The first (corresponding to the default for CCA is to optimize a single regularization parameters for both views (i.e. $c_1 = c_2$, 'coupled'). The second optimization ('separate') scheme corresponds with the method used in the main text and allows the regularization parameters to be different across views (i.e. $c_1 \neq c_2$). The first canonical weight vectors from these simulations are shown in Figure S3 (the second canonical weight vector shows similar behavior). When the when the views are balanced (Figure S3 A), both optimization schemes can accurately recover the true weights. In contrast, when the views are unbalanced (Figure S3 B and C), it is impossible for SCCA to accurately recover both weight vectors. Regularization parameters must be set such that either favour the first view at the expense of the second (Figure S3 B) or vice versa (Figure S3 C).

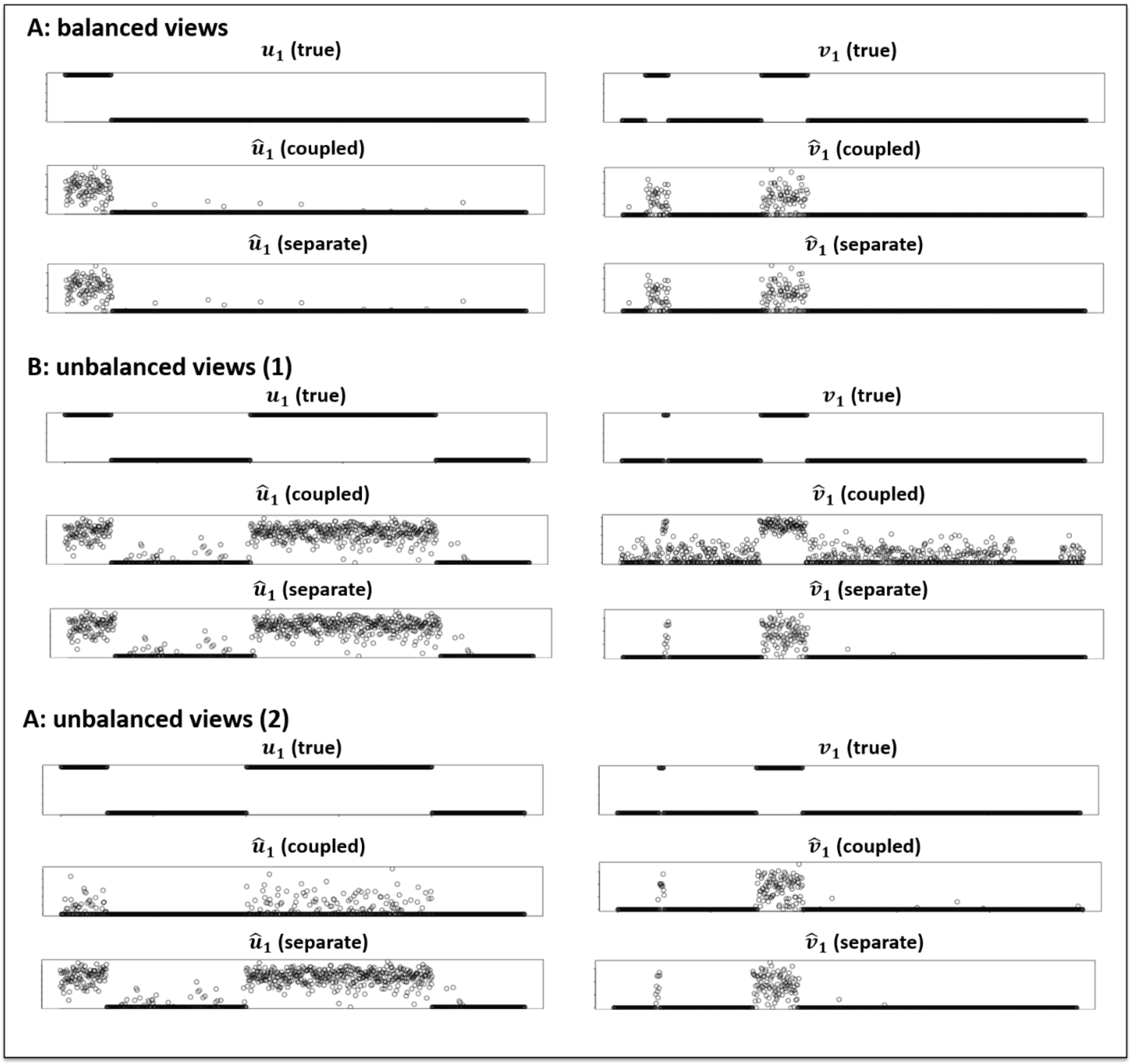

Figure S3: Results on the simulated data generated by the procedure shown in Figure S2. (A) when the number of non-zero features are balanced across views, both the basic SCCA algorithm with coupled regularization parameters (middle row) and the SCCA algorithm with separate regularization parameters used here (bottom row) do a good job of recovering the true weight vectors (top row). In contrast, when the number of non-zero features is unbalanced across views the basic SCCA algorithm can only recover one of the two weight vectors well, favouring either the first (u_1) at the expense of the second (v_1) (B) or vice versa (C). Note that only the weight vectors from the first component are shown.

Alternative visualization of SCCA weight vectors

To help summarize and visualize the canonical vectors in terms of anatomical brain regions we used the Automated Anatomical Labeling (AAL) atlas (Tzourio-Mazoyer et al, 2002) to obtain a normalized “anatomical signature” consisting of the weight per anatomical region per canonical vector (Figures 8 and 9). This weight is obtained by summing the values of the canonical vectors corresponding to the variables (voxels) within each anatomical region and dividing by the number of variables (voxels) in each region (Schrouff et al, 2013). To facilitate visualization, we have aggregated the weights for the left and right regions. Note that the weights are constrained to be positive so weights of different sign will not cancel one another. The anatomical weights for the first and second canonical vectors are shown respectively in Figures S4 and S5.

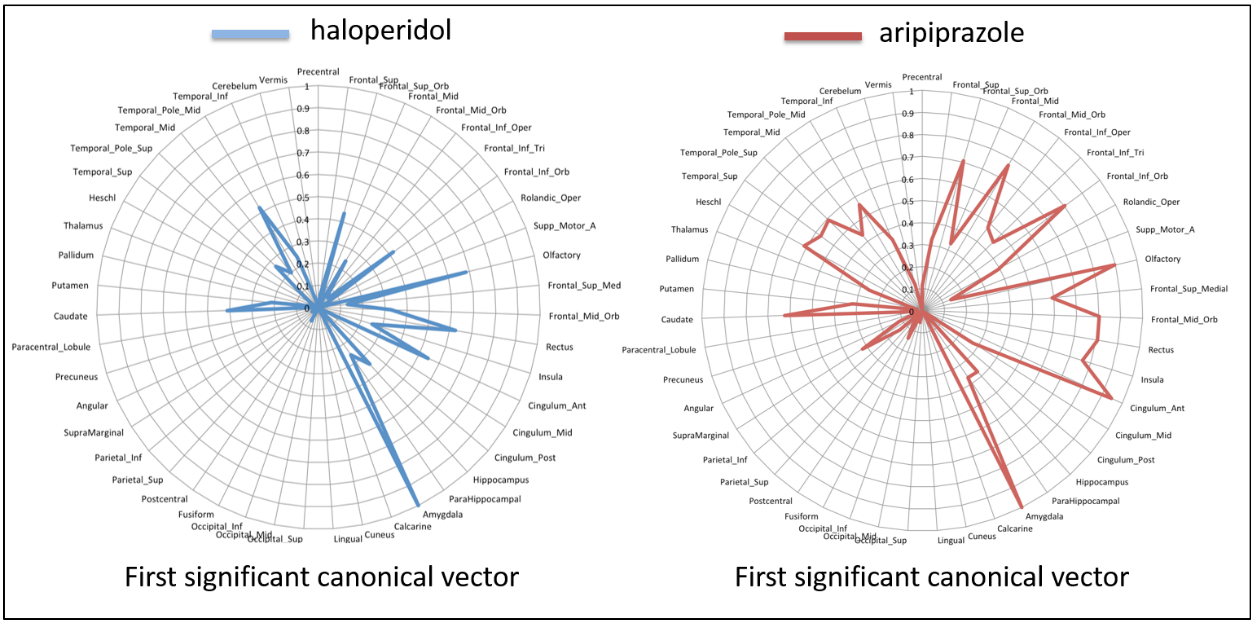

Figure S4: Normalized weights for the first set of canonical vectors for haloperidol and aripiprazole. The weights are obtained by summing the values of the canonical vector within an anatomical region defined by the AAL atlas and divided by the number of voxels inside this region.

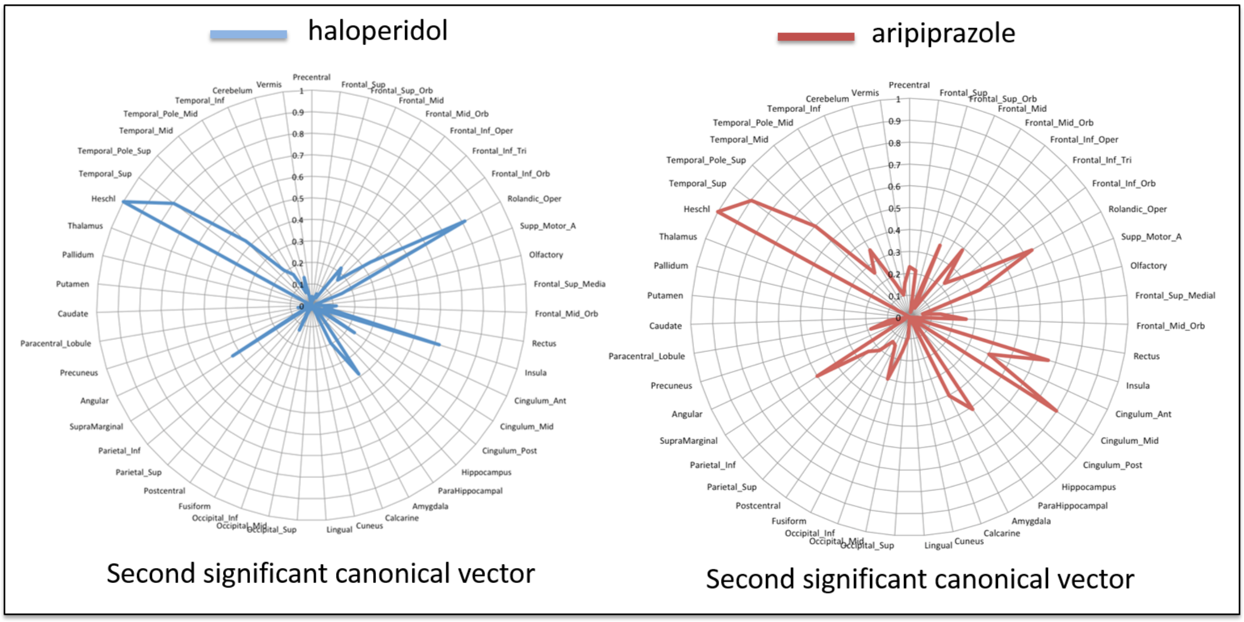

Figure S5: Normalized weights for the second set of canonical vectors for haloperidol and aripiprazole. The weights are obtained by summing the values of the canonical vector within an anatomical region defined by the AAL atlas and divided by the number of voxels inside this region.

Supplementary References

Schrouff, J., Cremers, J., Garraux, G., Baldassarre, L., Mourão-Miranda, J., & Phillips, C. (2013, June). Localizing and comparing weight maps generated from linear kernel machine learning models. In Pattern Recognition in Neuroimaging (PRNI), 2013 International Workshop on (pp. 124-127). IEEE.

Tzourio-Mazoyer, N., Landeau, B., Papathanassiou, D., Crivello, F., Etard, O., Delcroix, N., ... & Joliot, M. (2002). Automated anatomical labeling of activations in SPM using a macroscopic anatomical parcellation of the MNI MRI single-subject brain. Neuroimage, 15(1), 273-289.

Witten, D. M., Tibshirani, R., & Hastie, T. (2009). A penalized matrix decomposition, with applications to sparse principal components and canonical correlation analysis. Biostatistics, 10(3), 515-534.

Witten, D. M., & Tibshirani, R. J. (2009). Extensions of sparse canonical correlation analysis with applications to genomic data. Statistical applications in genetics and molecular biology, 8(1), 1-27.
